# Notch-RBP-J Signaling Regulates the Mobilization and Function of Endothelial Progenitor Cells by Dynamic Modulation of CXCR4 Expression in Mice

**DOI:** 10.1371/journal.pone.0007572

**Published:** 2009-10-27

**Authors:** Lin Wang, Yao-Chun Wang, Xing-Bin Hu, Bing-Fang Zhang, Guo-Rui Dou, Fei He, Fang Gao, Fan Feng, Ying-Min Liang, Ke-Feng Dou, Hua Han

**Affiliations:** 1 State Key Laboratory of Cancer Biology, Department of Medical Genetics and Developmental Biology, Xi-Jing Hospital, Fourth Military Medical University, Xi'an, China; 2 Department of Hepatic Surgery, Xi-Jing Hospital, Fourth Military Medical University, Xi'an, China; Leiden University Medical Center, Netherlands

## Abstract

Bone marrow (BM)-derived endothelial progenitor cells (EPC) have therapeutic potentials in promoting tissue regeneration, but how these cells are modulated in vivo has been elusive. Here, we report that RBP-J, the critical transcription factor mediating Notch signaling, modulates EPC through CXCR4. In a mouse partial hepatectomy (PHx) model, RBP-J deficient EPC showed attenuated capacities of homing and facilitating liver regeneration. In resting mice, the conditional deletion of RBP-J led to a decrease of BM EPC, with a concomitant increase of EPC in the peripheral blood. This was accompanied by a down-regulation of CXCR4 on EPC in BM, although CXCR4 expression on EPC in the circulation was up-regulated in the absence of RBP-J. PHx in RBP-J deficient mice induced stronger EPC mobilization. In vitro, RBP-J deficient EPC showed lowered capacities of adhering, migrating, and forming vessel-like structures in three-dimensional cultures. Over-expression of CXCR4 could at least rescue the defects in vessel formation by the RBP-J deficient EPC. These data suggested that the RBP-J-mediated Notch signaling regulated EPC mobilization and function, at least partially through dynamic modulation of CXCR4 expression. Our findings not only provide new insights into the regulation of EPC, but also have implications for clinical therapies using EPC in diseases.

## Introduction

Recently, bone marrow (BM)-derived cells, such as the hematopoietic stem cells (HSC), the mesenchymal stem cells (MSC), and the endothelial progenitor cells (EPC), have shown promising potential in the treatment of various human disease [1], [2]. For example, several reports have documented evidence that the transfusion of BM cells might benefit patients suffering the end-stage liver diseases, including those caused by liver cirrhosis, hepatitis virus B and hepatitis virus C infections, and alcohol abuse [3]–[8]. These studies showed that BM cells could contribute to liver regeneration after partial hepatectomy (PHx) [3], and reduce liver fibrosis in mouse disease models [4], [5]. Several groups have further shown the roles of EPC in liver regeneration and in the therapy of liver cirrhosis [6]–[8].

Since the discovery of EPC in the circulation of adults, great efforts have been made to characterize these cells and to show the roles of EPC in postnatal vasculogenesis and vessel repair [9]. EPC from BM or peripheral blood (PB) can be cultured and expanded in vitro [10]–[12], and bear the stem/progenitor cell markers like CD133, Sca-1, c-kit, and CD34, and the endothelial markers including Flk-1, CD31, vWF, UEA-1, and Tie-2 as well [13]. A large body of evidence has shown that EPC can be mobilized from BM and can home to wounded tissues [9]. EPC homed to the injury site can differentiate into endothelial cells (EC) to directly participate in vasculogenesis, and/or to produce angiogenic factors to contribute to vascular remodeling. Although these studies have prompted trials to use EPC to treat ischemic diseases [9] as well as to facilitate liver regeneration [6]–[8], signals regulating EPC mobilization and homing in vivo have been elusive. Among the molecules identified so far, such as angiogenic factors [14], integrins [15] and adhesion molecules [16], the chemokine receptor CXCR4-mediated signaling appears essential for EPC mobilization, migration, and differentiation [17]. SDF-1α, the ligand of CXCR4, is important in the trafficking and the homing of BM-derived cells including EPC [2], [18]–[21]. SDF-1α induced by hypoxia inducing factor-1α (Hif-1α) enhances the adhesion, migration, and homing of circulating CXCR4-positive EPC to ischemic tissues [20], [21]. But how SDF-1α-CXCR4 signaling is regulated in the mobilization and the recruitment of EPC to the injured tissues has been unclear.

Notch signaling represents a type of direct cell-cell communication that is essential for the regulation of proliferation, apoptosis, and fate decisions in stem/progenitor cells [22], [23]. Recently, Kwon et al found that Jagged-1 signaling from BM microenvironment was required for EPC development [24]. However, how the Notch signaling pathway exerts its roles in EPC has not been fully understood. The DNA-binding protein RBP-J (recombination signal binding protein-J▒) mediates signaling from all four mammalian Notch receptors by associating with the Notch intracellular domain (NICD), which is released upon the receptor triggering. This protein-protein interaction transactivates the genes downstream to RBP-J [22]. Notch signaling plays a pivotal role in the vascular system [23]. Using a *RBP-J* conditional knock-out mouse model, which phenocopies Notch deletion in multiple tissues, we recently showed that Notch signaling is critical in the maintenance of vascular homeostasis and EC-related functions [25], [26]. In this study, we report that Notch signaling regulates the mobilization and homing of EPC, probably by the dynamic modulation of CXCR4 expression.

## Results

### Mice transfused with the RBP-J deficient BM cells showed retarded SEC and hepatocyte regeneration after PHx

**Figure 1:**
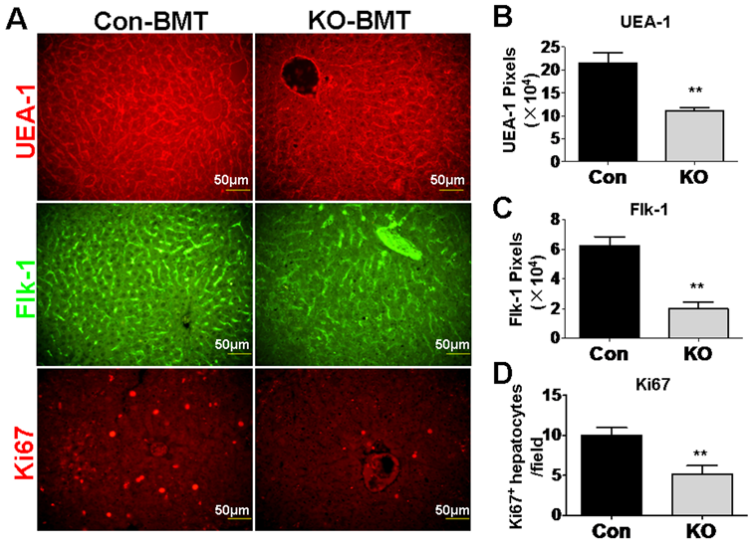
Mice transfused with the RBP-J deficient BM cells showed attenuated SEC and hepatocyte regeneration after PHx. BM cells were isolated from the RBP-J deficient or the control mice, and were transfused into irradiated normal mice. Two months later, the recipient mice were subjected to PHx, and were analyzed 3 days after the operation. (A) The immunofluorescent staining of UEA-1 (top), Flk-1 (middle), and Ki67 (bottom) with the liver sections of the recipient mice after PHx. (B, C) UEA-1- (B) and Flk-1- (C) positive signals in the upper and middle panels of (A) were quantified by the representative pixels, and were compared between the two groups. (D) Ki67^+^ round nuclei were counted under microscope, and were compared between the two groups. KO, RBP-J gene knockout mice; Con, control mice. Bars = mean±SD; n = 4; ** *P*<0.01.

To determine the role of the Notch-RBP-J signaling pathway in EPC, we employed the RBP-J conditional knockout mouse crossed with the Mx-Cre transgenic mouse, in which injection of poly(I)-poly(C) could induce almost 100% of RBP-J deletion in BM [27]. Fujii et al have shown that BM cells transfused into mice participate in liver regeneration after PHx, probably by commitment to SEC through EPC [3]. Therefore, we transfused BM cells from RBP-J deficient and control mice into normal irradiated mice. This BM transplantation led to more than 95% of blood cells of recipient mice derived from the donor BM cells 2 months later (result not shown). The mice transfused with the RBP-J deficient BM cells and the control BM cells were subjected to PHx, and the regeneration of SEC was evaluated on the third day after PHx, by immunofluorescent staining for Flk-1 and UEA-1. The result showed that the SEC regeneration was remarkably attenuated in the mice accepting RBP-J deficient BM cells compared to the mice accepting the control BM cells (Fig. 1A top and middle, and B, C). Moreover, we examined the proliferation of hepatocytes, a major mechanism of PHx-triggered liver regeneration. Ki67 staining demonstrated that proliferating hepatocytes (with Ki67^+^ round nuclei) were significantly less in mice transfused with RBP-J deficient BM cells (Fig. 1A bottom and D). These data suggested that mice with RBP-J-deleted BM cells were less efficient in liver regeneration than mice with normal BM cells, probably due to abnormal Notch signaling in EPC.

### Attenuated homing of the RBP-J deficient BM EPC into liver during liver regeneration

**Figure 2:**
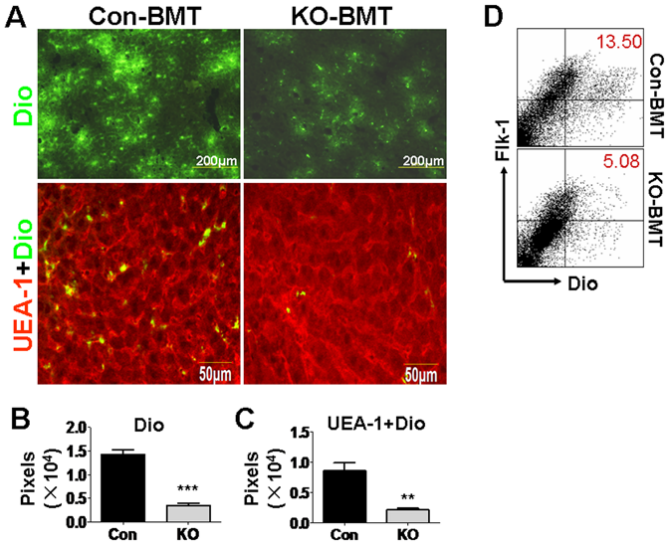
Lowered recruitment of the *RBP-J*-deleted EPC into the regenerating liver after PHx. Normal mice were subjected to PHx. On the next day of the operation, mice were irradiated, and were transfused with Dio-labeled BM cells from the RBP-J deficient (KO-BMT) or the control (Con-BMT) mice. The recipient mice were analyzed 2 days after the BM transplantation (3 days after PHx). (A) The livers of the recipient mice suffering PHx were sectioned, stained for UEA-1, and were examined for Dio^+^ cells (upper) and UEA-1^+^Dio^+^ cells (lower). (B, C) Dio^+^ cells and UEA-1^+^Dio^+^ cells in (A) were quantitatively represented by corresponding pixels (bars = mean±SD; n = 4; ** *P*<0.01; *** *P*<0.001). (D) Livers were perfused with PBS. SECs were purified from the livers of the recipient mice using a kit, and were analyzed for Flk-1^+^Dio^+^ cells. The cytograph represented 4 independent experiments with the same results.

We then compared the homing of the RBP-J deficient and the control EPC into the regenerating liver after PHx. Normal irradiated mice were subjected to PHx. On the second day of the operation, these mice were transfused with BM cells that were isolated from the RBP-J knockout mice or the control mice and were labeled with Dio. Two more days later (third day after PHx), mice were perfused, and Dio^+^ cells homed into the liver were examined under fluorescent microscope. As shown in Fig. 2A (upper) and B, BM cells from RBP-J deficient mice appeared to home into the regenerating liver less efficiently. We further performed immunofluorescence to evaluate the homing of RBP-J deficient EPC into the regenerating liver. The result showed that significantly less EPC or their derivatives (UEA-1^+^Dio^+^) were detected in the liver of mice accepting RBP-J deficient BM cells (Fig. 2A, lower, and C). SEC were collected from the regenerating liver using CD146-magnetic beads, and analyzed by FACS. The result showed that compared with the control, significantly less RBP-J deficient EPC or their derivatives (Dio^+^Flk-1^+^) were homed into the liver (Fig. 2D). These observations suggested that the blockade of Notch-RBP-J signaling in BM EPC retarded their homing to liver during liver regeneration after PHx.

### The dynamic change of the CXCR4 expression in the RBP-J deficient EPC

**Figure 3:**
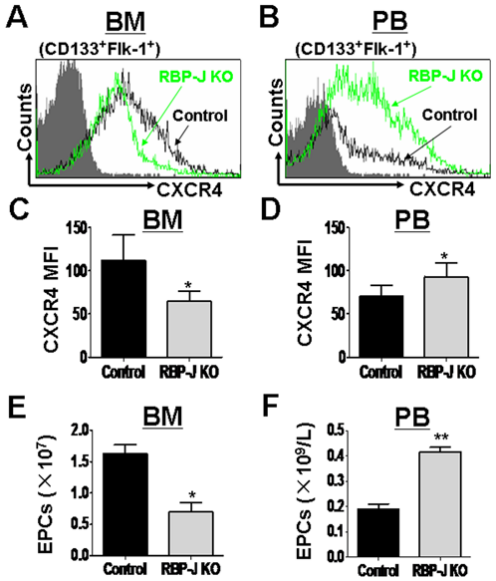
Disruption of *RBP-J* modulated the CXCR4 expression and the mobilization of EPC. Mice with different genotypes were induced to disrupt RBP-J, and BM and the peripheral blood (PB) cells were analyzed by FACS. (A, B) The expression of CXCR4 on CD133^+^Flk-1^+^ EPCs in BM (A) and PB (B) of the RBP-J deficient and the control mice was analyzed by FACS. (C, D) FACS analyses shown in (A) and (B) were repeated, and MFI of the CXCR4 expression was analyzed statistically (bars = mean±SD; n = 5; * *P*<0.05). (E, F) The number of CD133^+^Flk-1^+^ EPCs in BM (E) and PB (F) of the RBP-J deficient and the control mice was determined by cell counting and FACS analysis (bars = mean±SD; n = 5; *P<0.05; ** *P*<0.01).

SDF-1α-CXCR4 signaling has been shown to be the major signaling involved in the EPC mobilization. Injured tissue cells secret SDF-1α, the specific ligand for CXCR4, to chemotactically mobilize EPC from BM [2], [28]. We investigated the CXCR4 expression on EPC in BM and the peripheral blood of the RBP-J deficient and the control mice. The result showed that in BM, the CXCR4 expression on EPC was significantly decreased after RBP-J disruption (Fig. 3A and C). In contrast, the inactivation of RBP-J up-regulated the CXCR4 expression on EPC in PB (Fig. 3B and D). We examined the number of EPC in BM and PB of RBP-J knockout mice. As shown in Fig. 3E and Fig. 3F, the disruption of RBP-J led to the significant decrease of CD133^+^Flk-1^+^ EPC in BM, with a concomitant increase of EPC in PB. These results implied that more EPC were mobilized into the circulation. Therefore, the blockade of Notch-RBP-J signaling might result in autonomous mobilization of EPC from BM into PB, likely through the interference of CXCR4 expression. However, when we transplanted BM cells from the RBP-J deficient and the control mice into irradiated wild type mice, we did not observe significant difference in PB EPC or CXCR4 level between the RBP-J deficient group and the control group, suggesting that the mobilization of EPC into PB needs other signals.

### Inactivation of RBP-J increased EPC mobilization induced by PHx

**Figure 4:**
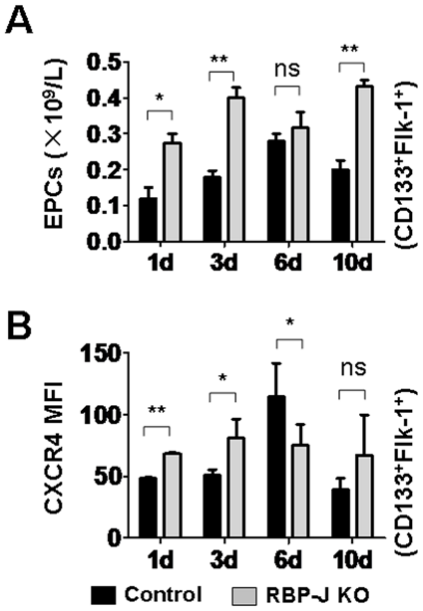
EPC mobilization in the *RBP-J* mutant and the control mice following PHx. The RBP-J deficient and the control mice were subjected to PHx. The number of EPC in PB (A) and the CXCR4 expression (B) on EPC in PB were analyzed by cell counting and FACS analysis at different time points (bars = mean±SD; n = 5; * *P*<0.05; ** *P*<0.01; NS, no statistical significance).

We then examined the PHx-induced EPC mobilization in the absence of the Notch-RBP-J signaling. The RBP-J knockout and the control mice were subjected to PHx, and EPC in PB were evaluated by FACS and cell counting. On day 1, 3 and 10 following PHx, more EPC were detected in PB of the RBP-J knockout mice as compared with the controls (Fig. 4A). The expression of CXCR4 on EPC was also up-regulated on these days after the PHx operation (Fig. 4B). The day 6 was special in that no significant difference in EPC level was detected between the RBP-J knockout and the control mice, while the expression of CXCR4 on the RBP-J knockout EPC was lower than that of the control EPC. These results suggested that the EPC mobilization was enhanced, correlating with the disturbed CXCR4 expression on EPC in the absence of RBP-J.

### RBP-J ablation damaged the functions of BM-derived EPC in vitro

**Figure 5:**
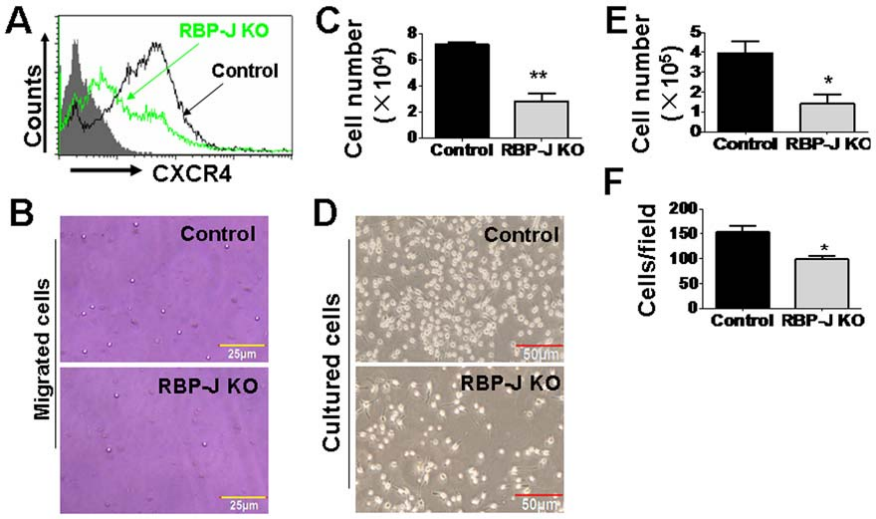
Effect of the RBP-J deletion on the in vitro cultured EPC. (A) The expression of CXCR4 on the cultured EPC was accessed by FACS analysis. (B, C) In vitro migration assay. Transwell culture was set up, with EPC from the RBP-J-deleted and the control mice in the upper chamber, and SDF-1α in the lower chamber. A photograph of EPC in the lower chamber (B) was taken, and cells in the lower chamber (C) were counted 18 h after the starting of the culture (bars = mean±SD; n = 4; ** *P*<0.01). (D) The formation of colonies by the cultured RBP-J knockout and the control EPC. (E) The number of total EPC formed 7 days after the culture. (F) The number of EPC attached on the gelatin-coated dishes 3 days after the culture. (bars = mean±SD; n = 4; * *P*<0.05).

Several properties, including adhesion and migration, are closely related to the functions of EPC in vasculogenesis [9], [24]. We then cultured BM EPC under the condition described in vitro [29]. We compared the CXCR4 expression on the cultured EPC, and found that CXCR4 expression was decreased upon the deletion of RBP-J (Fig. 5A). We then assessed EPC migration using a transwell assay. The result showed that RBP-J deficient EPC had a remarkably reduced migration capacity in response to SDF-1α (Fig. 5B and C). Therefore, RBP-J-disrupted EPC had lowered ability to migrate, probably due to their lowered CXCR4 expression. Moreover, the culture of RBP-J deficient BM generated decreased number of EPC (Fig. 5D and E) when compared with the control cultures, consistent with Kwon's results [24]. Cells attached to the gelatin-coated dishes on day 3 of the culture were counted, and the result showed that the adhesion ability of EPC was impaired in the absence of RBP-J (Fig. 5F).

### Impaired vessel formation in the RBP-J-deleted EPC was rescued by the over-expression of CXCR4

**Figure 6:**
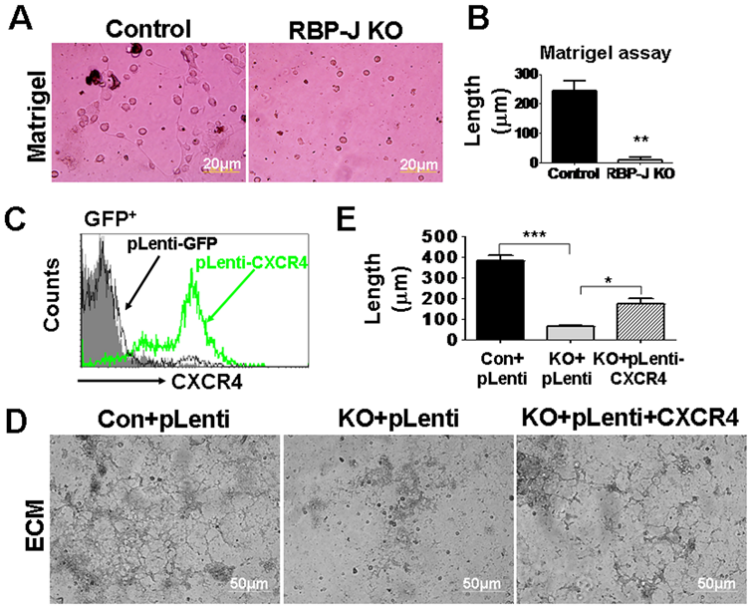
Over-expression of CXCR4 rescued the defects in vessel formation of the RBP-J deficient EPC. (A) Vessel formation by the cultured EPC with different genotypes in matrigel. (B) The quantitative analysis of vessel length in (A) (bars = mean±SD; n = 4; ** *P*<0.01). (C) The overexpression of the mouse CXCR4 by Lentivirus infection. HeLa cells were infected with different virons and the CXCR4 expression was assessed by FACS. (D) EPC with different genotypes were transduced with the Lentivirus vector or the Lentivirus expressing CXCR4, and were assayed for the vessel formation capability in the ECM culture system. (E) The quantification of the vessel length in (D) (bars = mean±SD; n = 4; * *P*<0.05; *** *P*<0.001).

EPC homed to the injury site can differentiate into EC to directly participate in vasculogenesis [9]. We compared the vessel formation ability between the RBP-J deficient and the control EPC. When cultured in Matrigel, EPC could form vessel-like structures (Fig. 6A). We found that the RBP-J knockout EPC could seldom form vessel-like structures when cultured in Matrigel (Fig. 6A and B). Similar defects in vessel formation were also detected when the RBP-J deficient EPC were cultured in ECM (Fig. 6D). Because the RBP-J deficient BM EPC had lowered CXCR4 expression, we transfected RBP-J knockout EPC with a Lentivirus expressing the mouse CXCR4 (Fig. 6C), and performed the vessel formation assay in ECM. The result showed that the over-expression of CXCR4 in EPC could partially rescue the defects in vessel formation of RBP-J deficient EPC (Fig. 6D and E). These results indicated that the Notch-RBP-J signaling was essential for the vessel formation by EPC, probably through CXCR4 expression.

## Discussion

The BM-derived EPC transplantation has been used in experimental therapy to facilitate liver regeneration. Transfused EPC can home to injured livers, and directly differentiate into SEC and integrated into liver sinusoids. They can also indirectly facilitate SEC and hepatocyte regeneration by paracrine mechanisms [30]–[32]. Our results suggested that the Notch signaling might play a role for the transfused BM-derived EPC to facilitate liver regeneration. When Notch signaling was interrupted, the BM-derived EPC appeared to have lower CXCR4 expression (Fig. 5A). Notch signaling is essential for these BM-derived EPC to incorporate into liver tissues and/or to participate in liver regeneration. Our results showed that the Notch signaling-mediated CXCR4 expression is necessary for EPC to participate in vessel formation. Furthermore, we have previously shown that RBP-J deficient SEC have attenuated capacity in supporting hepatocyte regeneration due to the lowered level of VEGFR1 expression [26]. These results suggested that the modification of EPC by the activation of their Notch signaling might be helpful to enhance the function of EPC in the therapy of liver diseases.

Although EPC have been shown to participate in liver regeneration [3] and might have therapeutic potentials in the treatment of end-stage liver diseases [6]–[8], how these cells are regulated in vivo has been poorly understood. The Notch-RBP-J signaling pathway is involved in the regulation of multiple lineages of stem/progenitor cells. In this study, using an inducible RBP-J knockout mouse model, we showed that Notch signaling might influence EPC at different levels or stages. In BM, the deficiency of the canonical Notch signaling led to the reduction of EPC, but a substantial fraction of EPC exists, suggesting that the Notch-RBP-J pathway is not essential for EPC commitment. Kwon et al reported a similar phenotype in the Notch ligand Jagged-1 knockout mice [24]. In the RBP-J deficient mice, however, we observed a concomitant increase of EPC in the peripheral blood. Because the RBP-J deficient EPC in BM showed lowered CXCR4 expression, we propose that the canonical Notch signaling represses EPC mobilization in BM by the maintenance of CXCR4 expression. In PB, RBP-J deficient EPC have up-regulated CXCR4, implying that Notch signaling might repress CXCR4 expression and EPC recruitment to injured tissues, since literature has indicated that the CXCR4 expression is closely related to the EPC recruitment into injured tissues, which produce SDF-1α [20], [21]. However, in the RBP-J knock-out model, although EPC in PB showed higher CXCR4 expression (Fig. 3 and 4), they were recruited into the regenerating liver (which produces SDF-1α) at a lower efficiency (Fig. 2). This suggests that additional Notch-regulated signals are also necessary for the homing of EPC from PB into the injured tissues, and further molecular studies are needed to identify these signals.

## Materials and Methods

### Ethnics Statements

The animal husbandry, experiments and welfare were conducted in accordance with the Detailed Rules for the Administration of Animal Experiments for Medical Research Purposes issued by the Ministry of Health of China, and were approved by the Animal Experiment Administration Committee of Fourth Military Medical University. Mice were raised in the specific pathogen free (SPF) conditions on the C57BL/6 background, and were manipulated with every specific care to reduce the suffering of the mice during the experiments.

### Mice

The *RBP-J*-floxed mice and the Mx-Cre transgenic mice were as described [27]. The *RBP-J*-floxed mice were crossed with the Mx-Cre mice to obtain heterozygous and homozygous mice bearing the Mx-Cre transgene (RBP^+/f^-MxCre and RBP^f/f^-MxCre, as the control and the RBP-J knockout mice, respectively), as genotyped by PCR [27]. The Cre-mediated deletion of *RBP-J* was induced by the intra-peritoneal injection of poly(I)-poly(C) (Sigma, St. Louis, MI) into 6-week-old mice with demanded genotypes, with a schedule descried in [27]. One week after the last injection, the mice were subjected to further analysis. PHx was performed as described [26].

### BM transplantation

The femurs and tibias of mice were dissected and flushed with phosphate-buffered saline (PBS). BM cells were resuspended at a density of 1×10^7^/ml. Wild type congenic mice as recipients were irradiated with 8 Gy γ-ray one day before the transfusion with 2×10^6^ BM cells via tail vein. In some experiments, cells were stained with Dio (Invitrogen, Carlsbad, CA) for 15 min, and were transfused into recipients. The mice were kept with water containing antibiotics (1.1 g/L of neomycin sulphate) until further analyses.

### Immunofluorescence

Liver embedded in OCT was sectioned at 10 µm thickness. For the staining, sections were fixed with 4% paraformaldehyde and were stained with FITC-conjugated anti-Flk-1 (Chemicon International), Rhodamine-UEA-l (Vector Laboratories, Burlingame, CA), or Cy3-conjugated anti-Ki67 (Santa Cruz Biotechnology, Santa Cruz, CA). Images were taken using a fluorescence microscope (Olympus BX51, Japan) with a CCD camera, or a confocal microscope (FV1000, Olympus).

### Flow cytometry

Single cell suspensions were prepared from cultured cells or freshly collected from PB, BM, or liver of mice. Following the treatment with buffered 0.14 M NH_4_Cl, cells (3−5×10^5^) were stained with antibodies for 30 min on ice, and were analyzed using a FACSCalibur^TM^ (BD Immunocytometry Systems, San Jose, CA). Data were analyzed using the CellQuest^TM^ software. Dead cells were excluded by propidium iodide (PI) staining. Anti-mouse-CD133-FITC, biotinylated anti-mouse-Flk-1, biotinylated anti-mouse-CD184 (CXCR4, 2B11) and streptavidin-APC were purchased from BD PharMingen (San Diego, CA).

### Isolation and culture of EPC

EPC were cultured as described previously [29]. Briefly, BM mononuclear cells were collected and cultured in 2% gelatin-coated 6 well dishes, using the Dulbecco's modified Eagle's medium (DMEM) containing 20% fetal calf serum (FCS), 50 µg/ml endothelial cell growth supplement (ECGS, Sigma), heparin (100 µg/ml) and antibiotics. Attached cells were allowed to develop into EPC for 7 days in the culture. EPC adhered to gelatin was evaluated by counting the attached cells on day 3 after the replacement of the medium. EPC were then analyzed by FACS or observed under a microscope on day 7.

### Migration assay

Chemotaxis experiments were performed in polycarbonate transwell inserts (5-µm pore, Corning Costar Corp.). SDF-1α (Peprotech) was added in the lower chamber at the concentration of 100 ng/ml. The cultured EPC (1×10^5^) were seeded in the upper compartment and were cultured at 37°C for 18 h. Migrated cells in the lower chamber were photographed and counted under a microscope.

### Vessel formation assay

Matrigel (BD Bioscience) or extracellular matrix (ECM, Sigma, St. Louis, MI) was added to 24-well plates in the volume of 300 µl and was allowed to solidify at 37 °C for 30 min. The equal volume of medium was added and was incubated for another 30 min. BM EPC (2×10^6^) were then seeded and were cultured with required supplements. After 7 to 10 days, the tube-shaped vessels in the Matirgel or ECM were photographed under a microscope.

### Lentivirus infection

The coding region of the mouse CXCR4 cDNA fragment was fused with the IRES-EGFP unit, and was inserted into pLenti-EGFP (kindly provided by Dr Xiao-Bing Wu) to replace the EGFP gene, to generate pLenti-CXCR4. pLenti-EGFP and pLenti-CXCR4 were transfected into the 293FT cells using Lipofectamine 2000, together with the packaging plasmids (Invitrogen), according to the manufacturer's protocols. The supernatants were collected 48 h after the transfection, and the virons were concentrated by ultracentrifugation at 70,000 g, at 4 °C for 2 h. The pellets were resuspended in DMEM and stored at −80 °C. For infection, EPC were co-cultured with the viral suspensions for 48 h, and were then cultured in normal medium until further analysis.

### Statistics

Images were imported into the Image Pro Plus 5.1 software, and the pixels for each color were analyzed to quantitatively represent the positively stained cells. Statistical analysis was performed with the SPSS 12.0 program. Results were expressed as means ± SD. Comparisons between groups were undertaken using unpaired Student's *t*-test. *P*<0.05 was considered statistically significant.

## References

[1] Houlihan DD, Newsome PN (2008). Critical review of clinical trials of bone marrow stem cells in liver disease.. Gastroenterology.

[2] Ueno T, Nakamura T, Torimura T, Sata M (2006). Angiogenic cell therapy for hepatic fibrosis.. Med Mol Morphol.

[3] Fujii H, Hirose T, Oe S, Yasuchika K, Azuma H (2002). Contribution of bone marrow cells to liver regeneration after partial hepatectomy in mice.. J Hepatol.

[4] Higashiyama R, Inagaki Y, Hong YY, Kushida M, Nakao S (2007). Bone marrow-derived cells express matrix metalloproteinases and contribute to regression of liver fibrosis in mice.. Hepatology.

[5] Sakaida I, Terai S, Yamamoto N, Aoyama K, Ishikawa T (2004). Transplantation of bone marrow cells reduces CCl4-induced liver fibrosis in mice.. Hepatology.

[6] Nakamura T, Torimura T, Sakamoto M, Hashimoto O, Taniguchi E (2007). Significance and therapeutic potential of endothelial progenitor cell transplantation in a cirrhotic liver rat model.. Gastroenterology.

[7] Taniguchi E, Kin M, Torimura T, Nakamura T, Kumemura H (2006). Endothelial progenitor cell transplantation improves the survival following liver injury in mice.. Gastroenterology.

[8] Beaudry P, Hida Y, Udagawa T, Alwayn IP, Greene AK (2007). Endothelial progenitor cells contribute to accelerated liver regeneration.. J Pediatr Surg.

[9] Urbich C, Dimmeler S (2004). Endothelial progenitor cells: characterization and role in vascular biology.. Circ Res.

[10] Sharpe EE,, Teleron AA, Li B, Price J, Sands MS (2006). The origin and in vivo significance of murine and human culture-expanded endothelial progenitor cells.. Am J Pathol.

[11] Kalka C, Masuda H, Takahashi T, Kalka-Moll WM, Silver M (2000). Transplantation of ex vivo expanded endothelial progenitor cells for therapeutic neovascularization.. Proc Natl Acad Sci U S A.

[12] Kawamoto A, Gwon HC, Iwaguro H, Yamaguchi JI, Uchida S (2001). Therapeutic potential of ex vivo expanded endothelial progenitor cells for myocardial ischemia.. Circulation.

[13] Asahara T, Kawamoto A (2004). Endothelial progenitor cells for postnatal vasculogenesis.. Am J Physiol Cell Physiol.

[14] Grunewald M, Avraham I, Dor Y, Bachar-Lustig E, Itin A (2006). VEGF-induced adult neovascularization: recruitment, retention, and role of accessory cells.. Cell.

[15] Chavakis E, Aicher A, Heeschen C, Sasaki K, Kaiser R (2005). Role of beta2-integrins for homing and neovascularization capacity of endothelial progenitor cells.. J Exp Med.

[16] Oh IY, Yoon CH, Hur J, Kim JH, Kim TY (2007). Involvement of E-selectin in recruitment of endothelial progenitor cells and angiogenesis in ischemic muscle.. Blood.

[17] Walter DH, Haendeler J, Reinhold J, Rochwalsky U, Seeger F (2005). Impaired CXCR4 signaling contributes to the reduced neovascularization capacity of endothelial progenitor cells from patients with coronary artery disease.. Circ Res.

[18] Petit I, Jin D, Rafii S (2007). The SDF-1-CXCR4 signaling pathway: a molecular hub modulating neo-angiogenesis.. Trends Immunol.

[19] Peled A, Petit I, Kollet O, Magid M, Ponomaryov T (1999). Dependence of human stem cell engraftment and repopulation of NOD/SCID mice on CXCR4.. Science.

[20] Yamaguchi J, Kusano KF, Masuo O, Kawamoto A, Silver M (2003). Stromal cell-derived factor-1 effects on ex vivo expanded endothelial progenitor cell recruitment for ischemic neovascularization.. Circulation.

[21] Ceradini DJ, Kulkarni AR, Callaghan MJ, Tepper OM, Bastidas N (2004). Progenitor cell trafficking is regulated by hypoxic gradients through HIF-1 induction of SDF-1.. Nat Med.

[22] Artavanis-Tsakonas S, Rand MD, Lake RJ (1999). Notch signaling: cell fate control and signal integration in development.. Science.

[23] Gridley T (2007). Notch signaling in vascular development and physiology.. Development.

[24] Kwon SM, Eguchi M, Wada M, Iwami Y, Hozumi K (2008). Specific Jagged-1 signal from bone marrow microenvironment is required for endothelial progenitor cell development for neovascularization.. Circulation.

[25] Dou GR, Wang YC, Hu XB, Hou LH, Wang CM (2008). RBP-J, the transcription factor downstream of Notch receptors, is essential for the maintenance of vascular homeostasis in adult mice.. FASEB J.

[26] Wang L, Wang CM, Hou LH, Dou GR, Wang YC (2009). Disruption of the transcription factor recombination signal-binding protein-Jkappa (RBP-J) leads to veno-occlusive disease and interfered liver regeneration in mice.. Hepatology.

[27] Han H, Tanigaki K, Yamamoto N, Kuroda K, Yoshimoto M (2002). Inducible gene knockout of transcription factor recombination signal binding protein-J reveals its essential role in T versus B lineage decision.. Int Immunol.

[28] Dalakas E, Newsome PN, Harrison DJ, Plevris JN (2005). Hematopoietic stem cell trafficking in liver injury.. FASEB J.

[29] Aoki M, Yasutake M, Murohara T (2004). Derivation of functional endothelial progenitor cells from human umbilical cord blood mononuclear cells isolated by a novel cell filtration device.. Stem Cells.

[30] Fausto N, Campbell JS, Riehle KJ (2006). Liver regeneration.. Hepatology.

[31] Matsumoto K, Yoshitomi H, Rossant J, Zaret KS (2001). Liver organogenesis promoted by endothelial cells prior to vascular function.. Science.

[32] LeCouter J, Moritz DR, Li B, Phillips GL, Liang XH (2003). Angiogenesis-independent endothelial protection of liver: role of VEGFR-1.. Science.

